# Effects of Ocean Acidification and Temperature Increases on the Photosynthesis of Tropical Reef Calcified Macroalgae

**DOI:** 10.1371/journal.pone.0154844

**Published:** 2016-05-09

**Authors:** Fernando Scherner, Cristiano Macedo Pereira, Gustavo Duarte, Paulo Antunes Horta, Clovis Barreira e Castro, José Bonomi Barufi, Sonia Maria Barreto Pereira

**Affiliations:** 1 Universidade Federal Rural de Pernambuco, Departamento de Biologia, R. Dom Manoel de Medeiros, s/n, Dois Irmãos, 52171–900, Recife, PE, Brazil; 2 Instituto Coral Vivo, Estrada da Balsa, km 4,5, Arraial d'Ajuda, 45816–000, Porto Seguro, BA, Brazil; 3 Universidade Federal do Rio de Janeiro, Museu Nacional, Departamento de Inverterbrados, Quinta da Boa Vista, s/n, São Cristóvão, 20940–040, Rio de Janeiro, RJ, Brazil; 4 Universidade Federal de Santa Catarina, Departamento de Botânica, Trindade, 88010–970, Florianópolis, SC, Brazil; 5 Universidade Federal de Pernambuco, Programa de Pós-graduação em Saúde Humana e Meio Ambiente, Rua Alto do Reservatório, s/n, 55608–680, Vitória de Santo Antão, PE, Brazil; University of Vigo, SPAIN

## Abstract

Climate change is a global phenomenon that is considered an important threat to marine ecosystems. Ocean acidification and increased seawater temperatures are among the consequences of this phenomenon. The comprehension of the effects of these alterations on marine organisms, in particular on calcified macroalgae, is still modest despite its great importance. There are evidences that macroalgae inhabiting highly variable environments are relatively resilient to such changes. Thus, the aim of this study was to evaluate experimentally the effects of CO_2_-driven ocean acidification and temperature rises on the photosynthesis of calcified macroalgae inhabiting the intertidal region, a highly variable environment. The experiments were performed in a reef mesocosm in a tropical region on the Brazilian coast, using three species of frondose calcifying macroalgae (*Halimeda cuneata*, *Padina gymnospora*, and *Tricleocarpa cylindrica*) and crustose coralline algae. The acidification experiment consisted of three treatments with pH levels below those occurring in the region (-0.3, -0.6, -0.9). For the temperature experiment, three temperature levels above those occurring naturally in the region (+1, +2, +4°C) were determined. The results of the acidification experiment indicate an increase on the optimum quantum yield by *T*. *cylindrica* and a decline of this parameter by coralline algae, although both only occurred at the extreme acidification treatment (-0.9). The energy dissipation mechanisms of these algae were also altered at this extreme condition. Significant effects of the temperature experiment were limited to an enhancement of the photosynthetic performance by *H*. *cuneata* although only at a modest temperature increase (+1°C). In general, the results indicate a possible photosynthetic adaptation and/or acclimation of the studied macroalgae to the expected future ocean acidification and temperature rises, as separate factors. Such relative resilience may be a result of the highly variable environment they inhabit.

## Introduction

Climate change is a key global threat to ocean ecosystems [1, 2]. Changes in ocean chemistry and seawater warming associated with increasing greenhouse gas concentrations have potential to affect the physiology of marine organisms [3] causing food webs changes [4] and leading to drastic shifts in marine communities [5]. The coming decades should present major ecological changes compared to the alterations that have already occurred [6]. According to the Intergovernmental Panel on Climate Change (IPCC) seawater warming and ocean acidification are still beginning in relation to the most negative scenarios forecasted by climatologists [7]. Thus, understanding the physiological and ecological effects of ocean acidification and increased temperatures on marine organisms is fundamental to predict future changes and assist with new management challenges of marine ecosystems.

Marine macroalgae are an important component of coastal ecosystems, supporting a high biodiversity and forming the base of marine food webs [8]. However, despite their great ecological importance, little attention has been given to the effects of climate change on macroalgae [6]. Calcifying macroalgae are common elements in tropical reefs, contributing significantly to the biogenic production of calcium carbonate (CaCO_3_) in reef systems [9, 10]. However, the experimental effort that has been devoted to understand the physiological responses of calcifying algae to ocean acidification and sea temperature increases is still relatively modest and focused mainly in coralline species, with little attention given to other important calcifying macroalgae groups. Thus, there is an urgent need for research on calcifying macroalgae responses to climate change [10].

Mixed responses by marine macroalgae to warming and acidification have been observed. It has been shown that some species benefit from higher CO_2_ levels despite the lower pH, enhancing the photosynthetic performance [11], while other species presented significant declines on photosynthesis [12, 13], and others have shown no significant alterations [14, 15]. Similarly, temperature increases have provided contrasting responses on photosynthesis of different macroalgae species [12, 16]. Such differences in the susceptibility of macroalgae to temperature rises and CO_2_-driven ocean acidification may drive species-level responses through adaptation, migration, and extinction [6], and have potential to substantially change marine coastal ecosystems. However, there are evidences that macroalgae inhabiting highly variable environments are relatively resilient to such changes [17]. Thus, evaluating the physiological effects of acidification and temperature is important to understand how calcifying macroalgae inhabiting a highly variable environment, such as the intertidal region, will respond to future changes. Although acidification and temperature are expected to interact with each other and with other factors, evaluating their physiological effects separately is a useful approach to understand the mechanisms of responses of these organisms to future changes.

Here we assessed the separate effects of ocean acidification and seawater temperature increases on the photosynthesis and carbonic anhydrase (CA) of macroalgae through experiments in mesocosm. We tested these factors on tropical calcifying macroalgae inhabiting the intertidal region, of which, three were frondose species representing the three major groups: the macroalgae *Halimeda cuneata* (Chlorophyta), *Padina gymnospora* (Phaeophyceae), and *Tricleocarpa cylindrica* (Rhodophyta), and a group of crustose coralline algae (Rhodophyta). Our null hypothesis is that increased temperatures and lower seawater pH, tested independently, do not cause alterations on the photosynthetic performance and CA of each tested macroalgae.

## Materials and Methods

### Experimental design and sampling procedures

Experiments were performed in a mesocosm facility located in Arraial d’Ajuda, a village in the municipality of Porto Seguro, Bahia, north-eastern Brazil (16°26'59" S, 039°03'53" W). The seawater used in the experiments was pumped continuously from a fringing reef situated 500 m from the mesocosm facility. Seawater was then kept in underground reservoirs to avoid excess heating. In these reservoirs seawater was treated accordingly (see below for details on temperature increases and CO_2_ addition). It was then pumped to elevated reservoirs, where seawater monitoring was performed before it reached the mesocosm tanks. The seawater flux rate was kept at three times the volume of the tanks per hour and was regulated on a daily basis. This system guaranteed that the seawater kept the same characteristics observed in the fringing reef, except for the treatments conditions. Water hydrodynamics within the tanks were provided by a re-circulation system installed in each tank (see [18] for more details). A shade cloth (Sombrite®) that reduced solar irradiation at 70% covered the area of the tanks. This way the irradiance in the tanks corresponded to the levels observed in the reefs under good conditions and at high tide, when depths were approximately 2.5m. This approach was necessary because previous evaluations without irradiation reduction detected bleaching and light-induced damage to PSII, probably because the tanks were shallow and tidal variations were not reproduced by the system.

The effects of acidification and increased temperatures were tested on three frondose calcareous macroalgae species: the Rhodophyta *Tricleocarpa cylindrica*, the Chlorophyta *Halimeda cuneata*, and the Phaeophyceae *Padina gymnospora*, and on a group of incrusting coralline species (Rhodophyta) that included *Lithophyllum stictaeforme*, *Pneophyllum conicum*, *Porolithon pachydermum*. However, in this study coralline crusts were measured and treated as a sole group. Macroalgae samples were collected from the lower intertidal region on the fringing reef situated in front of the mesocosm facility, with permissions obtained through “Sistema de Autorização e Informação em Biodiversidade–SISBIO” from ICMBio (Instituto Chico Mendes de Conservação da Biodiversidade). Four replicate tanks were used per treatment resulting in a total of 16 tanks, with treatments positioned randomly to assure environmental homogeneity among treatments. A plastic railing was positioned in the tanks to keep the macroalgae a certain level above the bottom of the tanks, minimizing the loads of sediment above them. Crustose corallines were placed with their living surface facing the bottom of the tanks to avoid excess irradiation. This way they were exposed to an average irradiance of 15 μmol photons m^−2^.s^−1^.

#### Acidification experiment

Acidification treatments consisted of enrichment of seawater with CO_2_ at different concentrations. The effects of these treatments were evaluated against control conditions, which consisted of seawater obtained from the same source, without any addition of CO_2_. This design represents the pH values relative to the current atmospheric CO_2_ levels of 400 ppm (pH approximately 8.1) and the predicted future conditions of acidified seawater corresponding to projected mid-century and late-century CO_2_ levels of 560 ppm (pH: approximately 7.85) and 1140 ppm (pH: approximately 7.60) under the A1F1 scenario by IPCC [7]. An additional extremely low pH level was set (approximately 7.2). This was particularly useful to investigate the acidification thresholds for the physiology of the selected macroalgae. Seawater pH was continuously monitored by the Reef Angel connected to pH probes and solenoid controllers, which assured permanent pH control, keeping it within an accepted range of desired pH levels (S1 Fig).

Macroalgae samples were kept on their natural substrata. Two specimens of each frondose species and four of coralline crusts were placed in each tank. Algae were acclimated in the tanks for 10 days before the experimental procedures began. The samples were kept in experimental conditions for 23 days. This experiment was carried out in November 2012.

#### Temperature experiment

To test the effects of increased seawater temperatures on macroalgae photosynthesis, three treatments of different temperatures above the natural seawater temperatures occurring in the seawater catchment area (~27°C) in March of 2012 were determined. The tested temperatures corresponded to the predicted increases in seawater temperatures based on three different scenarios expected by IPCC of 1, 2 and 4.5°C **[7]**. Temperatures varied according to natural variations occurring in the field **(S2 Fig)**. An automated system (Reef Angel Controller, Reef Angel) and 15.000W heaters, continuously controlled seawater temperatures, keeping seawater at the desired levels above control seawater. Temperature loggers (HOBO Water Temperature Pro v2 Data Logger—U22-001) were also installed at the elevated reservoirs.

Macroalgae samples were attached to plastic substrata. Three specimens of each species and coralline crusts were placed in each tank. Samples were acclimated in the tanks for 18 days before the experiment began and were exposed to treatments for 15 days afterwards.

### Photosynthesis measurements and analysis

The photosynthetic performance of macroalgae was measured using a Pulse Amplitude Modulated (PAM) fluorometer (Diving-PAM underwater fluorometer; Walz, Effeltrich, Germany) through the chlorophyll *a* fluorescence. Measurements were applied three times during the temperature experiment, after 4, 11 and 15 days from the beginning of the experiment. It was applied once during the acidification experiment, after 21 days on frondose algae and 23 days on coralline crusts. One measurement was applied on each pseudoreplicate totalizing three measurements on each species per tank for the temperature experiment, and two measurements on frondose algae and four measurements on coralline crusts per tank for the acidification experiment. To avoid pseudoreplication the average of the measurements of each tank were used for statistical analysis. The optimum quantum yield (*F*_v_/*F*_m_) of the macroalgae was obtained for the temperature experiment, while rapid light curves were applied for the acidification experiment, which allowed the further calculation of several photosynthetic parameters, as detailed below.

Measurements were applied using a ring shaped adaptor adjusted to keep a constant distance of 0.5 cm from the macroalgae to the fibre optic sensor. Samples were acclimated in the dark before readings for 30 minutes to obtain their maximal quantum yield. Equal acclimation periods were applied for each sample to avoid differences masked by repair mechanisms [19]. The basal fluorescence (*F*_o_) was obtained before a first saturating pulse (intensity = 8 PAM units during 0.8 s), which provided the maximal fluorescence emission by the samples. It allowed the calculation of the optimal quantum yield (*F*_v_/*F*_m_) (obtained as *F*_v_/*F*_m_ = (*F*_m_-*F*_o_)/*F*_m_). These procedures were followed by short applications of eight actinic light intensities for 5 s, followed by a short and strong saturating pulse of light, by applying the Rapid Light Curve (RLC) option of the Diving-PAM. Each saturating pulse of light allowed subsequent measurements of steady state fluorescence (*F*_t_) and maximum fluorescence (*F*_m_′) values. The effective quantum yield (Y(II)) was calculated from these values: Y(II) = (*F*_m_′-*F*_t_)/*F*_m_′ [20] and the relative electron transport rates (rETR) were accurately estimated: rETR = Y(II)*PAR*ETR-factor*FII. The ETR-factor applied was 0.84 for all species. The FII parameter is related to the proportion of chlorophyll *a* at the PSII, and its values were 0.8 for *P*. *gymnospora*, 0.5 for *H*. *cuneata*, and 0.15 for *T*. *cylindrica* and corallines [21]. RLC configurations were defined based on previous assessment for each species. PAR configurations used for frondose species ranged from 0–718 μmol photons m^−2^.s^−1^, while for coralline crusts it ranged from 0–486 μmol photons m^−2^.s^−1^, due to the great differences in light utilization between these groups. In addition to *F*_v_/*F*_m_, another descriptive parameter, the maximum electron transport rate (ETR_max_) were calculated by fitting each rETR curve to the Platt and Gallegos [22] function.

Complementary parameters were determined to investigate for possible alterations on the mechanisms of dissipation of energy caused by the treatments. The quantum yield of non-regulated non-photochemical energy loss in PS II (Y(NO)) and the quantum yield of regulated non-photochemical energy loss in PS II (Y(NPQ)) were calculated using Klughammer and Schreiber [23] modifications of Kramer et al. [24] dissipative parameter equations (/NO and /NPQ). Y(NO) corresponds to the fraction of energy that is passively dissipated as heat and fluorescence (mostly due to closed PSII reaction centers). Y(NPQ) reflects the fraction of energy dissipated as heat via the regulated photo-protective, non-photochemical quenching mechanism. These parameters were determined from *F*t, *F*m and *F*m’ values obtained after each saturating pulse, and calculated as Y(NO) = *F*t/*F*m and Y(NPQ) = (*F*t/*F*m’)–Y(NO). Additionally, the non-photochemical quenching (NPQ) was calculated based on the relationship between these two parameters: NPQ = Y(NPQ)/Y(NO). The definite integrals of the dissipative parameters and of Y(II) curves were calculated using the trapezoidal rule to allow comparisons among treatments [19].

### Carbonic anhydrase

The three species of frondose algae, cleared of epiphytes, were collected at the end of the acidification experiment and immediately stored in liquid nitrogen for further analysis. One sample from each tank was used as replicate totalizing four replicates per treatment. Weighted samples were ground in liquid nitrogen and extracted using 2 ml of a buffer containing 50mM Tris, 25 mM ascorbic acid and 5 mM EDTA added to 250 ml of distilled water. pH was adjusted to 8.5. The activity of CA was analysed potentiometrically at 0–2°C by measuring the linear pH drop time from 8.1 to 7.4 after adding 1 ml of ice-cold CO_2_-saturated H_2_O to the buffer-sample solution. Control consisted of measuring the pH drop time after adding CO_2_-saturated H_2_O to the buffer solution without macroalgae samples. A magnetic stirrer was used to keep the extract in suspension during measurements. The CA activity was obtained as REA (Relative Enzymatic Activity): REA = [(t_o_/t_c_)– 1] / FW, where t_o_ and t_c_ are the times pH has taken to drop within the determined range in control and enzymatic reactions, respectively; and FW is the final weight of the sample. This method was modified after [25].

### Statistics

Physiological effects on macroalgae were analysed using parametric statistics. After evaluating normality, photosynthetic data were tested for homogeneity of variances by the Cochran test. Because assumptions were met, Analyses of Variance (ANOVA) were performed. Repeated Measures ANOVA was applied using photosynthetic data of the temperature experiment, with temperature treatments as between-subject factor, and the time as within-subjects factor. One-Way ANOVA was performed for physiological data obtained from the acidification experiment, testing for significant differences among pH treatments. When significant differences were observed (p<0.05), the Newman–Keuls *post hoc* multiple comparison test was applied. All parametric statistical procedures were made using the data analysis software system STATISTICA 7.0 (StatSoft, Inc. 2004).

## Results and Discussion

### Acidification

#### Photosynthetic parameters

Coralline algae presented lower *F*_v_/*F*_m_ values at T(-0.9) comparing to the other treatments (Table 1; Table 2), while among frondose algae significant effects were observed only for *T*. *cylindrica*, which presented higher *F*_v_/*F*_m_ values at T(-0.9) in relation to T(0). ETR_max_ values presented no significant differences between treatments neither for coralline crusts nor for frondose species.

**Table 1:** Summary of ANOVA for photosynthetic parameters (*F*_v_/*F*_m_ e ETR_max_) and definite integrals obtained from curves of Effective Quantum Yield (Y(II)) and the dissipative parameters Y(NO), Y(NPQ) and Y(NPQ)/Y(NO) of the studied macroalgae species after 24 days of exposition to different acidification treatments. Values in bold represent significant differences (n = 4).

	Crustose Coralline Algae				*Tricleocarpa cylindrica*				*Halimeda cuneata*				*Padina gymnospora*			
	df	MS	F	p	df	MS	F	p	df	MS	F	p	df	MS	F	p
***F*_v_/*F*_m_**	**3**	**0.006**	**5.296**	**0.014**	**3**	**0.004**	**3.569**	**0.047**	3	0.008	1.702	0.219	3	0.0004	0.99	0.428
**ETR_max_**	3	0.004	0.243	0.864	3	0.052	0.554	0.655	3	1.069	0.856	0.49	3	7.012	2.52	0.107
**Y(II)**	3	2.122	0.218	0.881	3	21.52	0.443	0.726	3	34.74	0.538	0.664	3	121.28	3.03	0.07
**Y(NO)**	**3**	**494**	**4.14**	**0.031**	**3**	**2617**	**3.813**	**0.039**	3	333	1.28	0.325	3	629	0.88	0.478
**Y(NPQ)**	**3**	**481.9**	**4.7**	**0.021**	**3**	**2392**	**4.017**	**0.034**	3	96.01	2.373	0.121	3	218.3	0.53	0.673
**Y(NPQ)/Y(NO)**	**3**	**1696.3**	**5.341**	**0.014**	**3**	**5099**	**4.418**	**0.025**	3	119.5	2.29	0.13	3	564.6	0.63	0.608

**Table 2:** Photosynthetic parameters of the studied macroalgae species after 24 days of exposition to different acidification treatments (n = 4 ± SD). Different letters indicate significant differences according to Student Newman-Keuls post-hoc test (p <0,05).

		Treatments							
		T(0)		T(-0.3)		T(-0.6)		T(-0.9)	
**Crustose Coralline Algae**									
	*F*_v_/*F*_m_	0.47 ± 0.03	a	0.49 ± 0.03	a	0.47 ± 0.05	a	0.40 ± 0.03	b
	ETR_max_	0.73 ± 0.12	a	0.76 ± 0.10	a	0.68 ± 0.13	a	0.71 ± 0.17	a
***Tricleocarpa cylindrical***									
	*F*_v_/*F*_m_	0.50 ± 0.05	a	0.55 ± 0.05	ab	0.56 ± 0.03	ab	0.62 ± 0.01	b
	ETR_max_	1.22 ± 0.41	a	1.21 ± 0.42	a	0.97 ± 0.13	a	1.15 ± 0.14	a
***Halimeda cuneata* **									
	*F*_v_/*F*_m_	0.55 ± 0.02	a	0.50 ± 0.05	a	0.44 ± 0.09	a	0.49 ± 0.09	a
	ETR_max_	4.66 ± 0.52	a	4.88 ± 0.73	a	4.32 ± 1.80	a	3.70 ± 0.98	a
***Padina gymnospora***									
	*F*_v_/*F*_m_	0.65 ± 0.02	a	0.65 ± 0.02	a	0.65 ± 0.02	a	0.67 ± 0.02	a
	ETR_max_	11.15 ± 1.49	a	9.60 ± 1.22	a	11.03 ± 1.68	a	12.83 ± 2.15	a

**Fig 1:**
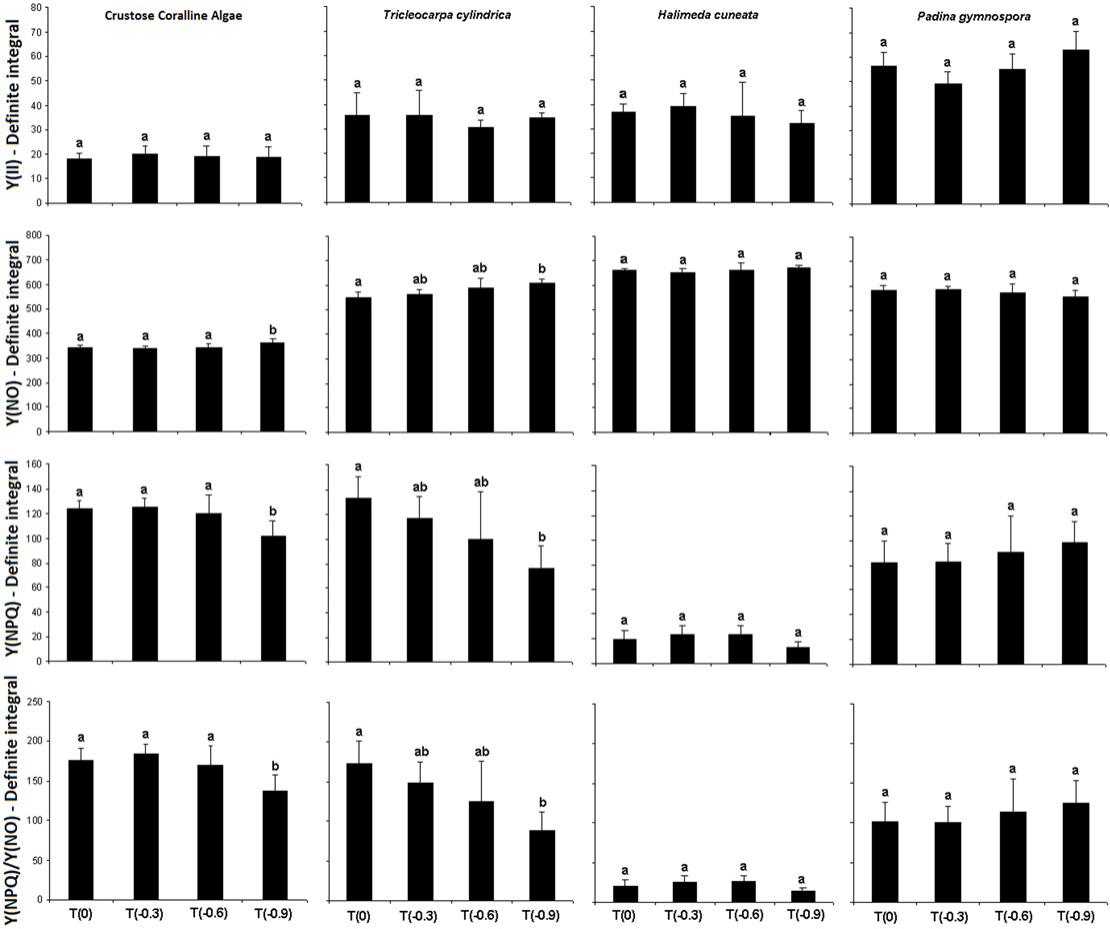
Definite integrals of Effective Quantum Yield (Y(II)) and the dissipative parameters Y(NO), Y(NPQ) e Y(NPQ)/Y(NO) obtained from the studied macroalgae species after 24 days of exposition to different acidification treatments (n = 4 ± SD). Different letters above bars indicate significant differences according to Student Newman-Keuls post-hoc test (p <0.05).

The definite integrals obtained from Y(II) curves presented no differences among treatments for any algae (Fig 1 and Table 1). Considering the dissipating mechanisms, significant differences between treatments on definite integrals values from their respective curves were observed only for coralline algae and *T*. *cylindrica*. The former group presented higher non-regulated energy dissipation (Y(NO) values) and lower Y(NPQ) and NPQ values at T(-0.9) comparing to the other treatments. Similarly, *T*. *cylindrica* presented higher Y(NO) values and lower Y(NPQ) and NPQ values at T(-0.9) but only in relation to T(0) (Fig 1).

Significant effects of acidification on the photosynthetic performance of the studied macroalgae were observed to coralline algae and *T*. *cylindrica*, which presented significant alterations at pH levels -0.9 below the current natural values observed in the studied region. At this condition, an interesting trend emerged with the former group presenting declines on *F*_v_/*F*_m_ values, while the latter species showed higher *F*_v_/*F*_m_ values. Despite this opposite trend on *F*_v_/*F*_m_, both presented higher Y(NO) and lower Y(NPQ) and NPQ. These parameters represent different fates of excitation energy in PSII, providing information on the organisms’ capacity to adapt to excess excitation energy under different conditions. Considering that Y(II) represents the fraction of energy that is photo-chemically transformed in PSII, the residual energy corresponds to the total quantum yield of all loss processes. This remaining portion can be divided into two components: Y(NO) and Y(NPQ). Increased Y(NO) values indicate a lower ability of the seaweed to protect itself against photodamage, while increased Y(NPQ) values reflect enhanced photo-protective capacities [23]. Thus, our results indicate a decrease in photo-protective capacities of coralline algae and *T*. *cylindrica* at -0.9 reduced pH.

In spite of the negative effects observed on the photosynthesis of coralline algae and *T*. *cylindrica* at the lowest tested pH, the expected pH declines for the 21^st^ century are within the range of 0.6 even at the most negative scenarios [26]. Our results indicate no effects on the light reactions phase of photosynthesis on any of the tested macroalgae within this range of pH values, as opposed to the expected increase of photosynthetic rates by fleshy macroalgae under elevated CO_2_ [27]. The patterns observed here suggest that photosynthetic rates are saturated by current inorganic carbon (Ci) composition of seawater. However, literature reports are still inconclusive in this regard, with some studies showing enhanced photosynthesis of numerous macroalgae species with increased dissolved inorganic carbon [27], while others observed no changes in photosynthetic performance [14, 15]. Israel and Hophy [14] suggest that the CO_2_ concentrating mechanisms (CCMs) commonly present in marine macroalgae are the likely reason for the insensitive photosynthetic responses of macroalgae to elevated CO_2_. Most macroalgae use HCO_3_^-^ for photosynthesis, with a few exceptions, regardless of the division [27]. This ability to acquire HCO_3_^-^ is important to increase carbon acquisition in seawater, where CO_2_ diffusion is limited. However, there are evidences that the fleshy chlorophyte *Ulva* sp. increased the use of CO_2_ compared to HCO_3_^-^ at CO_2_ enrichment conditions [28]. In a future scenario of elevated CO_2_ in the oceans, increases in the total dissolved inorganic carbon (DIC) and shifts on the relative proportion of different DIC species should occur. In this scenario, the proportion of CO_2_ will have the greatest increase among DIC species of over 250%, while HCO_3_^-^ pool will increase by only 24%, even though in absolute figures it will rise more than CO_2_ [27]. Thus, this ability to change the proportion of dependence to each DIC species according to the new high CO_2_ conditions provides an important alternative at a high CO_2_ scenario because it minimises energy allocation used in the process of carbon acquisition, resulting in a possible competitive advantage [27].

#### Carbonic anhydrase

**Fig 2:**
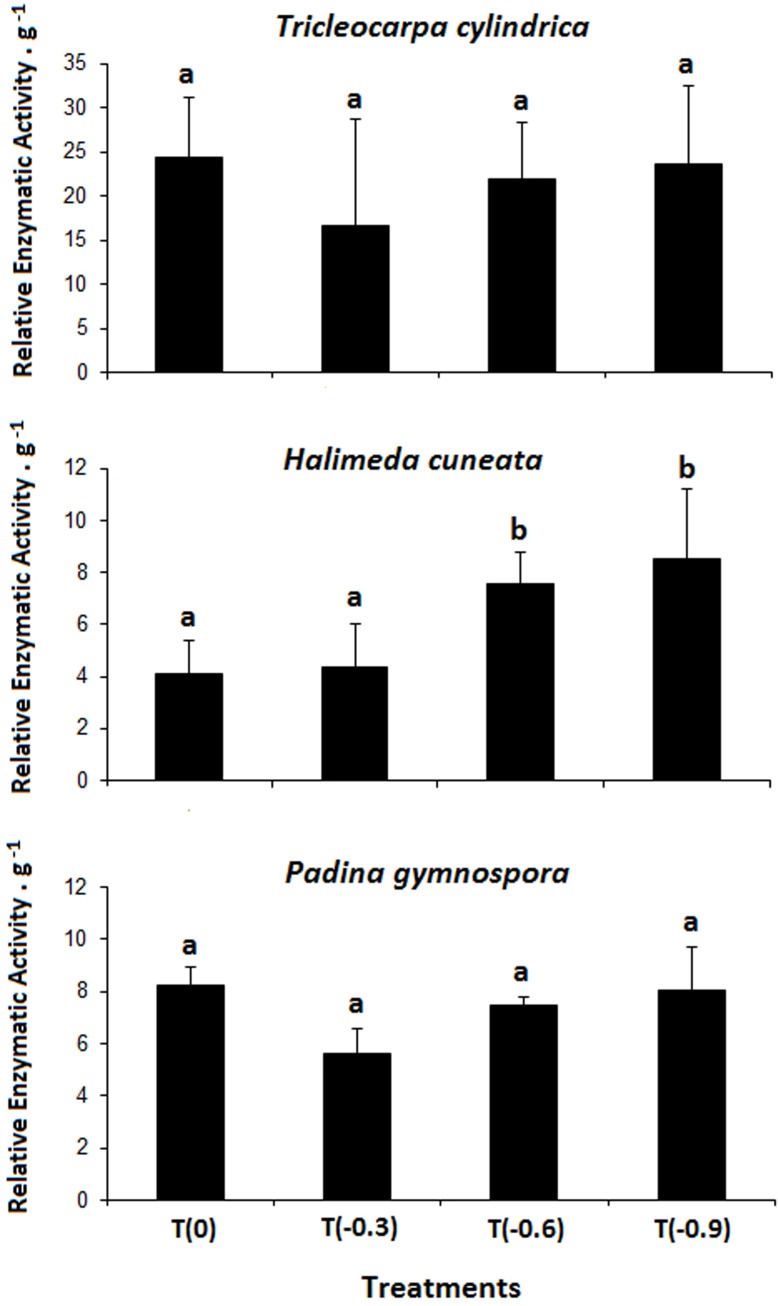
Relative Enzymatic Activity of Carbonic Anhydrase obtained from the studied macroalgae species after 24 days of exposition to different acidification treatments (n = 4 ± SD). Different letters above bars indicate significant differences according to Student Newman-Keuls post-hoc test (p <0.05).

The CA activity presented different trends according to the macroalgae species. *H*. *cuneata* presented significant higher CA values at T(-0.6) and T(-0.9) in relation to T(0) and T(-0.3) (F = 6.02; p < 0.01), while *T*. *cylindrica* and *P*. *gymnospora* showed no significant effects (p>0.05) of treatments on CA activity (Fig 2).

The increased CA values observed for *H*. *cuneata* at the two lower pH treatments (T(-0.6) and T(-0.9)), comparing to T(0) and T(-0.3), indicates a supply of HCO_3_^-^ for direct uptake over the plasma membrane through hydration of CO_2_ [29]. It is possible that the increase in CA values was triggered by the disproportional increase in availability of CO_2_ in relation to HCO_3_^-^ that occurs at highly CO_2_ enriched conditions. As proposed by Wizemann et al. [30], photosynthesis, external carbonic anhydrase activity, and cell-uptake of ions are tightly coupled in *Halimeda*. HCO_3_^-^ from the seawater is used by *Halimeda* spp. for both photosynthesis and calcification. External CA activity catalyzes H_2_O and CO_2_ to HCO_3_^-^and protons (H^+^), which are necessary for nutrient and HCO_3_^-^/H^+^ symport into the cell [30]. Thus, it is likely that external CA was responsible for the increased values observed in *H*. *cuneata* in our study. The other two frondose species showed little sensitivity to low pH conditions, suggesting a lower dependence of CA for Ci uptake.

Although in this study calcification was not quantified, many studies have shown that rates of coralline algae calcification diminish with acidification (e.g. [31–33]). Calcification in calcified algae such as *Halimeda* and coralline species has been closely associated to photosynthesis in a way that a deficit of one leads to declines of the other [30, 34–37]. The rate of ocean acidification should also have an important role on photosynthetic responses of coralline algae, as it is to their structure [38]. The possible ecological consequences of decreased calcification include reduced abundance of these algae due to higher vulnerability to herbivory and reduced capacity of attachment in areas with high hydrodynamics.

### Temperature

**Fig 3:**
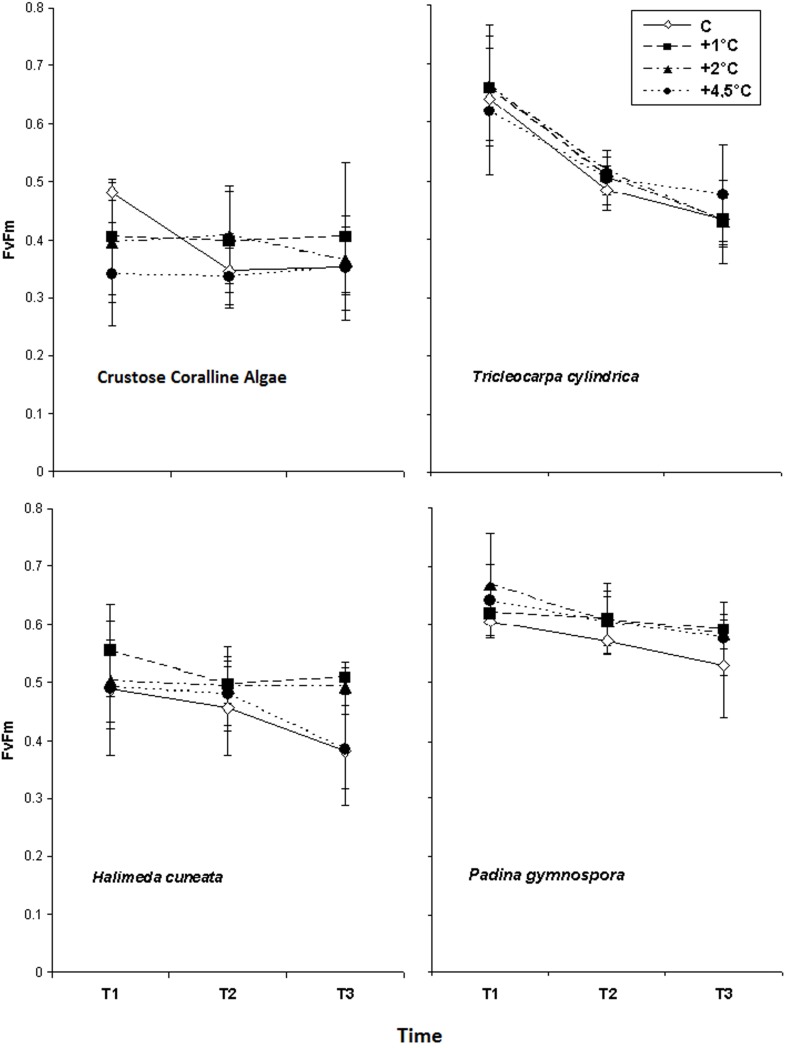
Average Optimum Quantum Yield (*F*_v_*/F*_m_) obtained from the studied macroalgae species in response to different temperature increasing treatments obtained through three measurements (T1, T2 and T3), after 4, 11 and 15 days of exposition (n = 4 ± SD).

The coralline algae, *T*. *cylindrica*, and *P*. *gymnospora* showed no effects of higher temperatures on *F*_v_/*F*_m_ along the experimental period. *H*. *cuneata* presented significantly higher *F*_v_/*F*_m_ values only at the treatment +1°C in relation to control and +4.5°C treatments (Table 3 and Fig 3)

**Table 3:** Summary of Repeated Measures ANOVA for Optimum Quantum Yield (*F*_v_/*F*_m_) obtained from macroalgae exposed to increased temperatures treatments. Values in bold represent significant differences (p<0.05) as shown by Student Newman-Keuls (SNK) post-hoc test (n = 4), (T = Time).

		df	MS	F	p	SNK
**Crustose Coralline Algae**		** **	** **	** **	** **	
** **	Temperature	3	0.008	0.71	0.55	
** **	Time	2	0.006	1.65	0.21	
** **	Time*Temperature	6	0.006	1.59	0.19	
***Tricleocarpa cylindrica***						
*** ***	Temperature	3	0.0007	0.25	0.86	
*** ***	Time	**2**	**0.17**	**34.45**	**0**	T3 < T2 < T1
*** ***	Time*Temperature	6	0.0018	0.369	0.89	
***Halimeda cuneata***						
*** ***	Temperature	**3**	**0.016**	**5.6**	**0.012**	+1°C > C, +4.5°C
*** ***	Time	2	0.018	2.95	0.07	
*** ***	Time*Temperature	6	0.003	0.624	0.7	
***Padina gymnospora***						
*** ***	Temperature	3	0.0058	1.65	0.220	
	Time	**2**	**0.016**	**6.68**	**0.004**	T3 < T1
	Time*Temperature	6	0.0008	0.33	0.91	

The lack of significant photosynthetic responses to rises in temperature by coralline algae, *T*. *cylindrica* and *P*. *gymnospora* are in accordance with other studies [16] supporting the adaptability of many species to variations in temperature. However, *H*. *cuneata* presented enhanced *F*_v_/*F*_m_ values at modest temperature increases, which is not surprising due to its tropical nature, occurring conspicuously in tropical regions [39, 40], where such variations are within the normal range. Despite this affinity to moderate higher temperatures, an increase to 4.5°C above control seawater, to an average of 31°C, led to lower *F*_v_/*F*_m_ values than those observed at inferior temperature increases. Although these values were still comparable to those obtained from control samples, it indicates that higher temperature increases reverses the positive responses achieved at modest temperature increases. This is supported by other study on *H*. *macroloba* and *H*. *cylindracea*, which have observed significant declines on F_v_/F_m_ values of both species at 32°C [12]. It suggests that biogeographical shifts may occur for *Halimeda* species from low to higher latitudes if temperature increases above the species limits for adequate physiological performance, disappearing from excessively warm areas and occupying new areas where temperature should become suitable. However, it has been suggested that ocean acidification should introduce additional physiological stresses that could lead to smaller overall thermal ranges available for new occupations [41].

The environment the species and/or specimen inhabit may have an important role on how they cope climate change. Egilsdottir et al., [17] have shown that articulated coralline algae inhabiting highly variable environments such as rocky pools present robust physiological responses to ocean acidification, which may be a result of adaptation and/or acclimation [17]. This may be a likely explanation for the general insensitivity observed with the tested species in our study, considering that all of the specimens we used were collected from the intertidal region, a very variable environment. Additionally, our experimental design was set to evaluate the physiological responses of selected macroalgae to ocean acidification and temperature separately. This is a useful approach to understand the effects of each factor on the physiology of the organisms, allowing different levels of each factor to be tested using a robust design. However, the results obtained from this perspective should be interpreted conservatively because the processes of warming and ocean acidification interact with each other. Such synergism leads, for instance, to a greater effect on coralline algal mortality comparing to the impacts of separate factors [26].

## Conclusions

Overall, our experiments have shown little effects of acidification and temperature rises on the tested species and groups. With regard to acidification, our null hypothesis was confirmed for *P*. *gymnospora*. Significant effects were observed in some photosynthetic parameters in *T*. *cylindrica* and coralline algae, although only at the extreme acidification treatment (-0.9), which is beyond the expected range of change. Only *H*. *cuneata* presented changes in CA activity. Regarding the temperature rises, our null hypothesis was rejected only for *H*. *cuneata*. Significant effects of the temperature experiment were limited to an increased photosynthetic performance by this species although only at a modest temperature rise (+1°C). Since the highest tested temperature showed no difference from the control, this effect was difficult to interpret. In general, the results suggest a possible photosynthetic adaptation and/or acclimation of the studied macroalgae to the expected future ocean acidification and temperature rises, as separate factors. Such relative resilience may be a result of an adaptation to the highly variable environment they inhabit, the intertidal region.

## Supporting Information

S1 Fig Average of 24 hours pH variations of different treatments during the experimental period of the acidification experiment gathered by the Reef Angel system. Gray and white arrows represent moments when measurements of photosynthesis were performed for frondose and coralline algae, respectively.
(JPG)

S2 Fig Average hourly temperature variations of different treatments during the experimental period of the temperature experiment gathered by the Reef Angel system. Arrows represent moments when measurements of photosynthesis were performed. Black bars on top represent gaps on measurements due to calibration and/or replacements of sensors.
(JPG)
