# Supplementary figures and images for: Effects of Ocean Acidification and Temperature Increases on the Photosynthesis of Tropical Reef Calcified Macroalgae
Source: PLoS One. 2016 May 9;11(5):e0154844. doi: 10.1371/journal.pone.0154844 (PMC4861303; doi:10.1371/journal.pone.0154844)

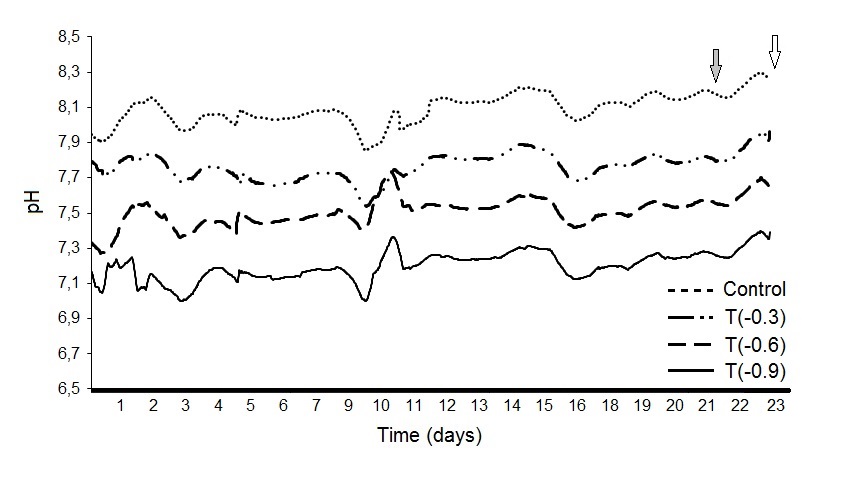

Supplement: S1 Fig — Gray and white arrows represent moments when measurements of photosynthesis were performed for frondose and coralline algae, respectively. (JPG) [file pone.0154844.s001.jpg]

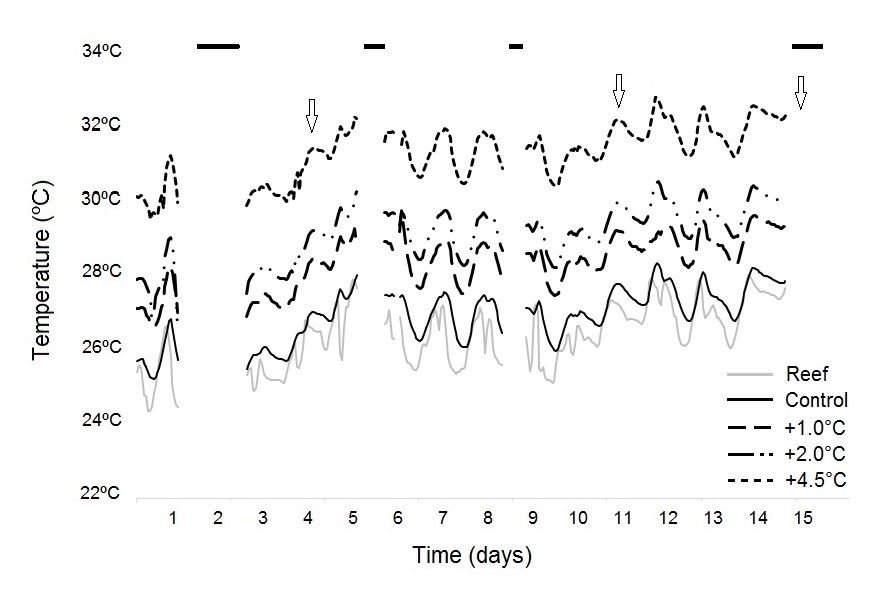

Supplement: S2 Fig — Arrows represent moments when measurements of photosynthesis were performed. Black bars on top represent gaps on measurements due to calibration and/or replacements of sensors. (JPG) [file pone.0154844.s002.jpg]
